# Targeting Inhibitor of Apoptosis Proteins to Overcome Chemotherapy Resistance—A Marriage between Targeted Therapy and Cytotoxic Chemotherapy

**DOI:** 10.3390/ijms241713385

**Published:** 2023-08-29

**Authors:** Tiago Barroso, Cecília Melo-Alvim, Leonor Abreu Ribeiro, Sandra Casimiro, Luís Costa

**Affiliations:** 1 Medical Oncology Department, Hospital de Santa Maria, Centro Hospitalar Universitário Lisboa Norte, 1649-035 Lisbon, Portugal; cecilia.alvim.moreira@gmail.com (C.M.-A.); lcabreuribeiro@gmail.com (L.A.R.); luiscosta.oncology@gmail.com (L.C.); 2 Luís Costa Lab, Instituto de Medicina Molecular João Lobo Antunes, Faculdade de Medicina da Universidade de Lisboa, 1649-028 Lisbon, Portugal; scasimiro@medicina.ulisboa.pt

**Keywords:** cancer, apoptosis, XIAP, cIAP1, cIAP2, xevinapant, tolinapant

## Abstract

Precision oncology is the ultimate goal of cancer treatment, i.e., to treat cancer and only cancer, leaving all the remaining cells and tissues as intact as possible. Classical chemotherapy and radiotherapy, however, are still effective in many patients with cancer by effectively inducing apoptosis of cancer cells. Cancer cells might resist apoptosis via the anti-apoptotic effects of the inhibitor of apoptosis proteins. Recently, the inhibitors of those proteins have been developed with the goal of enhancing the cytotoxic effects of chemotherapy and radiotherapy, and one of them, xevinapant, has already demonstrated effectiveness in a phase II clinical trial. This class of drugs represents an example of synergism between classical cytotoxic chemo- and radiotherapy and new targeted therapy.

## 1. Introduction

Precision oncology is the ultimate goal of cancer treatment, i.e., to treat cancer and only cancer, leaving all the remaining cells and tissues as intact as possible. The term “precision oncology”, while ironically imprecise according to some authors [1], speaks to the desire to maximize effectiveness while minimizing toxicity. Classical cytotoxic chemotherapy, while effective, is plagued by severe side effects affecting most organ systems and sometimes a cumulative toxicity profile that limits the duration of its usage. This is an important drawback in metastatic solid tumors, where a cure is not a possibility, and treatment must be given continuously to avoid disease progression while preserving quality of life. The antitumor antibiotics anthracyclines are a classic example; while effective in a number of tumor types, their use in metastatic disease is limited due to dose-dependent cardiac toxicity [2,3].

By acting on tumor-specific mechanisms, targeted therapy comes with a promise of effectiveness (i.e., the drug is toxic for cancer cells) with minimal toxicity (i.e., the drug has very low toxicity to healthy cells and tissues). Probably one of the greatest examples of target therapy is the development of tyrosine kinase inhibitors for chronic myeloid leukemia, a hematological disease invariably dependent on a given chromosome translocation, t(9;22)(q34;q11), leading to survival gains measured in decades with relatively low toxicity, such that the life expectancy of patients with a new diagnosis of chronic myeloid leukemia is now similar to the general population [4]. However, this promise is not always realized; even a drug that is maximally specific for a molecular target essential for tumor growth and survival can cause side effects on healthy cells in which the same molecular mechanism is important (on-target off-tumor side effects). An example of on-target off-tumor toxicity is the cardiac toxicity of the HER2 receptor antagonists used in breast cancer, which may cause idiosyncratic (i.e., non-dose dependent) heart failure by acting on HER2 receptors in the heart [5]. Also, target drugs might not be completely specific for a given mechanism, thus leading to side effects via their effect on similar mechanisms (off-target off-tumor side effects). Moreover, unless the targeted mechanism is absolutely essential for tumor survival and proliferation, treatment resistance may develop. Such resistance can be either intrinsic or acquired. A classic example is the mutation in KRAS in colon cancer, which confers intrinsic resistance as well as acquired resistance to EGFR inhibitors. An increase in the proportion of KRAS-mutated cells can be documented via serial molecular testing from a liquid biopsy of circulating cells throughout treatment with EGFR inhibitors [6].

Classical antineoplastic chemotherapy works primarily by causing DNA damage [7]. DNA damage leads to the activation of downstream mechanisms, which can either result in DNA repair (and consequent cell survival), programmed cellular death (known as apoptosis), or senescence [8,9]. Senescent cells do not replicate and are resistant to chemotherapy. In the metastatic setting, where total and long-lasting eradication of measurable disease is often impossible, therapy-induced senescence is probably a worthwhile goal. However, senescent cells are still alive and can influence the tumor microenvironment via the release of oncogenic molecules [10,11,12,13]. The utility of inducing senescence is demonstrated by the success of cyclin-dependent kinase 4/6 (CDK4/6) inhibitors in metastatic breast cancer, which actively promotes senescence, with benefits in both progression-free survival and overall survival [14,15,16]. However, in the setting of localized disease, where a cure is desired, the role of senescence is not as clear. The use of abemaciclib (a CDK4/6 inhibitor) for 2 years after surgery for locally advanced breast cancer has shown efficacy in increasing disease-free survival at 4 years [17]. However, long-term follow up is still required to show how sustained this response is. Conversely, due to the disadvantages of senescence described above, an alternative approach is to avoid senescence. Senolytics are a heterogeneous group of drugs that attempt to avoid or reverse senescence when used together with other cancer treatments. They are defined as drugs capable of killing senescent cells. BCL-2 inhibitors (discussed below), as well as the drug combination of dasatinib + quercetin (an association of tyrosine kinase inhibitors), may be considered senolytics. The pre-clinical evidence for the use of senolytics is mixed, and the role of this class of drugs is not yet clear [18].

Despite the importance and promise behind modern targeted therapy, one must not forget that cytotoxic chemotherapy is still very effective in the treatment of cancer. A large retrospective systematic literature review has examined patient eligibility for chemotherapy and the effectiveness of several chemotherapy schemes. The authors estimated that in the population of advanced or metastatic cancer patients from the United States, 78% were eligible for some kind of cytotoxic chemotherapy. Among those, the average objective response rate was 48% [19]. It is clear, then, that cytotoxic chemotherapy is still an essential tool for modern oncologists in the era of targeted treatment. However, just like in targeted therapy, resistance to chemotherapy tends to develop throughout treatment, which in metastatic disease leads inevitably to disease progression, loss of quality of life, and death. When chemotherapy is used as part of a curative strategy (as in neoadjuvant chemotherapy, adjuvant chemotherapy, and concomitant chemo- and radiotherapy), resistance to chemo- and radiotherapy can lead to local or distant cancer relapse. Two paradigmatic examples are squamous cell cancer of the head and neck and cervical cancer. The high failure rates and significant morbidity of surgery in the upper aerodigestive tract and pelvic cavity make the use of definitive chemo- and radiotherapy an attractive possibility.

The use of novel targeted agents to overcome chemotherapy and radiotherapy resistance without the need to increase chemotherapy dose or add new cytotoxic drugs to a given treatment protocol is an enticing possibility and one that is just now bearing fruit in the form of a positive phase II randomized clinical trial [20], with ongoing phase III trials [21]. These treatments sit at the intersection between classical cytotoxic chemotherapy and a deep understanding of the biology of cell death, growth, and survival. One such treatment is the drug xevinapant, a first-in-class non-specific inhibitor of three inhibitors of apoptosis proteins (cIAP1, cIAP2, and XIAP), which sensitizes cancer cells to the cytotoxic effects of chemotherapy and radiotherapy-induced DNA damage.

In this paper, we review the theoretic underpinnings behind the effectiveness of xevinapant, as well as other apoptosis inducers such as birinapant and tolinapant, focusing on xevinapant, which has already shown clinical benefit in early-phase studies [22]. Then, we briefly review the pre-clinical data that supports the use of cIAP1/2 and XIAP inhibitors in clinical trials. In the end, we will review the clinical results and ongoing trials with this class of drugs and outline future areas of research for this class of drugs based on the current understanding of the role of apoptosis and the IAPs.

## 2. Mechanisms of Action of Chemotherapy and Radiotherapy

Classical cytotoxic or cytostatic chemotherapy works by inhibiting either cellular division or by causing direct DNA damage. Similarly, radiotherapy works by damaging DNA directly and promoting cellular stress [8]. DNA can be damaged via various mechanisms, namely oxidative damage, the alkylation of bases, base loss caused via the hydrolysis of the bases, bulky adduct formation, DNA crosslinking, and DNA strand breaks, consisting of single and double breaks. Unless DNA damage can be repaired by the cell via the normal endogenous DNA repair mechanisms, the damaged DNA will lead to the induction of apoptosis mainly through the intrinsic pathway [23].

**Figure 1:**
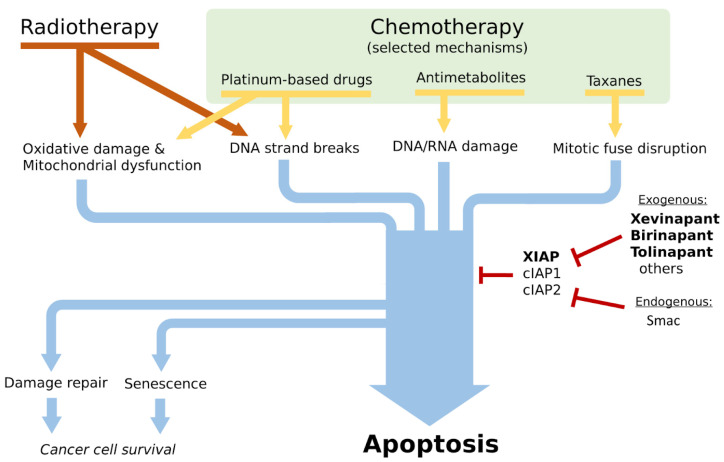
Selected mechanisms of action of chemotherapy and radiotherapy and the role of inhibition of apoptosis proteins (IAPs) and their physiological (Smac) and pharmacological (xevinapant, birinapant, tolinapant, others) inhibitors. Based on [8,24]. Abbreviations: Smac—second mitochondrial activator of caspases, IAP—inhibitor of apoptosis proteins, cIAP1 and cIAP2—cellular inhibitor of apoptosis proteins 1 and 2, XIAP—X-linked inhibitor of apoptosis protein.

As an illustrative example, one can look at the mechanisms of action of some of the following common chemotherapy drugs: the platinum-based drug cisplatin, the antimetabolite 5-fluorouracil, and the anti-cytoskeletal agents of paclitaxel and docetaxel. Cisplatin is incorporated into the cell, where it induces DNA damage (single- and double-strand break) in both nuclear and mitochondrial DNA. It can also bind to structures in the cytoplasm, leading to reactive oxygen species. 5-fluorouracil works by being converted into active metabolites, which are mistakenly incorporated in DNA and RNA during nucleic acid replication (as if they were normal nucleotides), leading to DNA and RNA damage. The taxanes paclitaxel and docetaxel bind to microtubules in the mitotic fuse, which inhibits mitotic cell division. The common end result of all these three mechanisms is the induction of cell death via apoptosis [24]. Similarly, radiotherapy induces DNA damage and oxidative stress similar to cisplatin, leading to apoptosis via similar mechanisms. Indeed, in the concomitant use of chemo- and radiotherapy, one sees a synergic effect when cisplatin is used as a radiosensitizing agent [8]. The overexpression in IAPs is a viable strategy for the tumor to resist treatment-induced cell death, which suggests that the use of inhibitors of these proteins might bring clinical benefit (Figure 1).

## 3. Apoptosis Pathways in Health and Disease

Cells are extremely efficient chemical reactors, optimized for breaking down and building up several essential molecules, among which are those molecules that compose the cells themselves. Thus, cells contain in themselves the mechanisms for their own destruction. The organized program for cell death is named apoptosis, as opposed to the main form of unprogrammed cell death, named necrosis. Like most essential biological processes, apoptosis is tightly regulated via the interaction of both pro-apoptotic and anti-apoptotic mechanisms. Once the threshold for apoptosis is reached, the process proceeds through a cascade in which molecules activated at a given step catalyze the amplification of the apoptotic process via a positive feedback mechanism. This is analogous to the feedback loop in other biologically important cascades, such as the coagulation cascade. A full description of the known mechanisms behind apoptosis in eukaryotic cells is beyond the scope of this present review and can be revisited in a dedicated review [25].

Two main pathways for apoptosis have been described: the intrinsic and the extrinsic pathways. As the name suggests, the intrinsic pathway is activated via mechanisms that come from the inside of the cell, namely DNA and mitochondrial damage. The extrinsic pathway, however, is triggered when an outside mechanism orders the cell to die, usually via signaling from extracellular receptors. Current cancer therapy exploits the induction of apoptosis through both of these mechanisms, cytotoxic chemotherapy, and radiotherapy, by acting on cell division and/or damaging DNA directly, attempting to trigger apoptosis through the intrinsic pathway, while anti-PD1/PD-L1 and anti-CTLA4 immunotherapy, for example, act by inhibiting immune tolerance and allow cytotoxic lymphocytes to trigger the extrinsic pathways via the stimulation of extracellular death receptors. Other target therapies act via a number of mechanisms that cause cell death [24].

Among the endogenous inhibitors of apoptosis, we will focus on the family of inhibitors of apoptosis proteins (IAPs). In humans, eight proteins have been described in this family: NAIP, cellular IAPs (cIAP1 and cIAP2), the X-linked IAP (XIAP), Survivin, Bruce/Apollon, ML-IAP/Livin, and ILP-2 [26]. The most well studied are cIAP1, cIAP2, and XIAP, and of these, XIAP is the most potent inhibitor of apoptosis [27]. These proteins share several common functional domains, which act in different ways to inhibit apoptosis [26,27].

**Figure 2:**
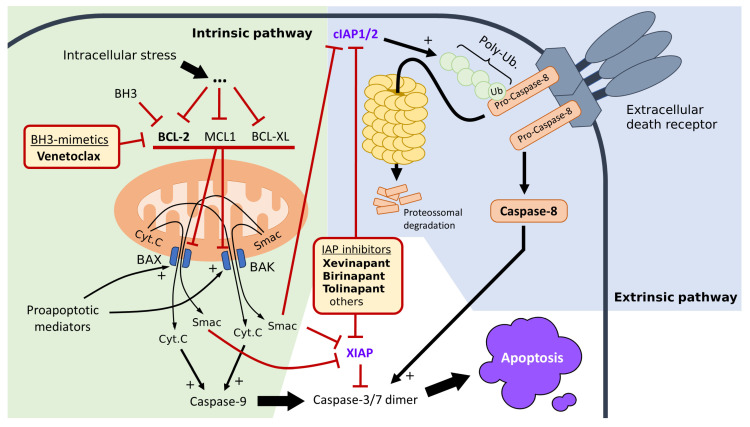
The intrinsic pathway of apoptosis (left-hand side of the figure, highlighted in light green) is triggered via intracellular stress, such as DNA damage triggered via chemotherapy or radiotherapy. The mitochondria are fundamental in this process, as later steps depend on the release of pro-apoptotic molecules (cytochrome C and Smac) via the mitochondria. The release of cytochrome C and Smac (and, by extension, apoptosis) is prevented via proteins of the BCL family, such as BCL-2, MCL1, and BCL-XL. These apoptosis inhibitors can be inhibited via the endogenous BH3 or an exogenous analog, such as the BH3-mimetic venetoclax. The proteins of the BCL family inhibit the BAX and BAK channels, which regulate the permeability of the mitochondrial membrane. Once this inhibition is lifted, BAX and BAK increase the membrane permeability, leading to the release of cytochrome C and Smac, which activates the caspase cascade. This process can be inhibited via XIAP, which directly inhibits the Caspase 3/7 dimer further downstream. The extrinsic pathway of apoptosis (right-hand side of the figure, highlighted in pale blue) is triggered via extracellular death receptors of the TNF superfamily, such as Apo2L/TRAIL and Fasl. The activation of these receptors via extracellular ligands leads to the activation of pro-Caspase 8 into its active form (Caspase 8). The cIAP1/2 proteins are able to poly-ubiquitinate Caspase 8 (as well as other pro-apoptotic proteins) and target them for degradation in the proteasome. Not pictured here are the remaining 5 IAPs, namely NAIP, Survivin, Bruce/Apollon, ML-IAP/Livin, and ILP-2. The reader is directed to [29,30] for a more detailed treatment of how those IAPs integrate with the apoptosis pathways. Both pathways culminate in the formation of the activated Caspase-3/7 dimer, which triggers apoptosis via a number of effector mechanisms. Abbreviations: Smac—second mitochondrial activator of caspases, Cyt.C—cytochrome C, IAP—inhibitor of apoptosis proteins, cIAP1/2—cellular inhibitor of apoptosis protein 1 and 2, XIAP—X-linked inhibitor of apoptosis protein, Ub—ubiquitin, Poly-Ub—poly-ubiquitination, BH3—BCL-2 homology domain 3, MCL1—Myeloid cell leukemia 1, BCL-XL—B-cell lymphoma-extra large, BCL-2—B-cell lymphoma 2, BAK—BCL-2 antagonist/killer, BAX—BCL-2-associated X protein.

As shown in Figure 2, in both the intrinsic and extrinsic apoptotic pathways, the terminal event is the activation of the caspase-3 and caspase-7 complexes. The IAPs can block caspase activation via two main mechanisms: they can act as enzyme blockers, blocking substrate entry into the caspase active domain, or target the caspases for proteasomal degradation via the process of ubiquitination [26]. Ubiquitination is an evolutionarily conserved process to promote the degradation of proteins tagged with the ubiquitin peptide [28]. In both mechanisms, the end result is a functional decrease in caspase activity and, thus, a greater degree of apoptosis inhibition. Biochemical studies show that XIAP inhibits caspase function mainly by binding to the catalytic site, thus preventing substrate entry. In contrast, the cIAPs inhibit caspase activity by adding ubiquitin to caspases such as caspase-8, thus targeting them for degradation in the proteasome [26].

Current evidence suggests cIAP1 and cIAP2 are more relevant in the inhibition of apoptosis triggered via the extrinsic pathway, while XIAP is more relevant in the intrinsic pathway, although significant overlap exists [20,26,27].

Besides the IAPs, another important family of apoptosis inhibitors is the BCL-2 (B-cel-lymphoma 2) family of proteins. Those proteins inhibit the apoptosis upstream of the IAPs in the intrinsic pathway. They inhibit the release of the pro-apoptotic molecules Smac and cytochrome C. In the same way, the IAPs have endogenous inhibitors; the BCL-2 family can be inhibited via the BH3 family of molecules. The exogenous inhibitors of members of the BCL-2 family have been developed, and some, such as venetoclax, have shown effectiveness in clinical trials [31].

The IAPs have pleiotropic effects on other pathways of cell death and survival, including the modulation of the nuclear factor κB (NF-κB) pathway. These effects relate to the immune response, cell survival, cell proliferation, or motility [32]. One should note these exact processes are dysregulated in biological and clinically meaningful ways in cancer: an immune-suppressive tumor environment with dysregulated inflammation promotes (pathological) immune tolerance, cell proliferation leads to tumor bulk increase, a higher burden of disease, and abnormal motility leads to invasion and metastatic spread. Currently, it is not clear what are the exact contributions of several IAPs for each of these processes.

As is typical of multi-layered biological systems, the IAPs have inhibitors themselves. Among those is the second mitochondrial activator of caspases (Smac). Drugs have been developed that mimic the biological function of Smac, and as such have been named Smac-mimetics. Examples of such drugs are xevinapant (AT-406 or Debio 1143, developed by Debiopharm and now licensed to Merck, Lausanne, Switzerland), birinapant (TL32711, developed by Tetralogic Corp., Malvern, PA, USA), and tolinapant (ASTX660, Astex Pharmaceuticals, Cambridge, UK). These drugs inhibit cIAP1, cIAP2, and XIAP to varying degrees. For example, both xevinapant and birinapant have a higher affinity for cIAP1 when compared to XIAP [33,34]. Still, the biological relevance of this selectivity is not yet completely clear, as both XIAP and cIAP 1/2 are relevant in preclinical models of cancer.

## 4. Inhibition of Apoptosis Proteins in Cancer Biology and Treatment

**Figure 3:**
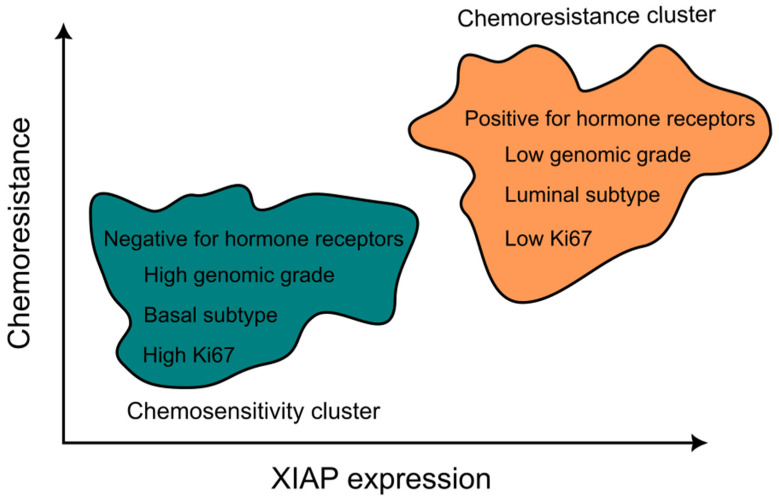
Conceptual illustration of the relationship between XIAP expression and phenotypic markers of breast cancer (according to the results from the bioinformatics analysis of RNA and protein expression in breast cancer cells) which are classically associated with either chemotherapy resistance or chemotherapy sensitivity. Based on [36].

Current pre-clinical data are quite consistent in identifying XIAP overexpression in tumors as a prognostic factor of poor survival and chemo- and radioresistance [27,35,36]. The evidence for cIAP1 and cIAP2 as poor prognostic markers is not as consistent, but they seem important in certain cases [37,38]. However, a question arises on whether XIAP is simply a marker of tumor aggressiveness or, more specifically, a marker of chemoresistance. A recent study on breast cancer [36] may shed some light on this question. When comparing two subtypes of breast cancer, with luminal and basal phenotypes, the luminal subtype exhibits characteristics such as hormone sensitivity, lower levels of cellular replication with more often indolent time course, and a less immunogenic phenotype. The basal subtype is notable for its often very aggressive behavior, with high levels of cellular replication as measured using the Ki67 marker, a more immunogenic subtype, such that phase III clinical trials have shown the effectiveness of immunotherapy in metastatic disease [39] and in neoadjuvant and adjuvant treatment [40]. In the metastatic setting, survival is longer in luminal disease (overall survival approximately 5 years when CDK4/6 inhibitors with hormone therapy are used in the first or second line) [15,17] when compared to triple-negative disease (currently 25 months with first-line concomitant immunotherapy and chemotherapy) [39]. However, despite their less aggressive time course and sensitivity to hormone therapy, luminal breast cancers are less sensitive to the cytotoxic effects of chemotherapy when compared to the more aggressive basal breast cancer. A recent bioinformatic analysis of publicly available breast cancer databases, including mRNA and protein expression, has shown that a number of breast cancer characteristics classically associated with chemosensitivity or chemoresistance (molecular grade, hormone receptor expression, luminal vs. basal subtype, and proliferative index) are associated with low or high XIAP expressions, respectively (Figure 3) [36]. These preclinical data suggest that XIAP might indeed work as a prognostic marker not by tracking tumor aggressiveness but by correlating with apoptosis and chemosensitivity directly.

Besides the direct effects of apoptosis, the IAPs seem to regulate inflammation, the immune response, and cell migration. Pre-clinical studies have shown that the IAP inhibitors are radio- and chemo-sensitizing in isolated cells, lab-grown organoids, and human tumors implanted in live mice. Concretely, IAP expression (in particular XIAP) seems to modulate the tumor microenvironment, and conversely, the tumor microenvironment seems to regulate IAP expression in tumor cells [22,34,41,42]. This evidence stemmed from ongoing early-phase clinical trials in which IAP inhibitors are combined with immunotherapy for solid tumors, namely the ASTEROID trial with tolinapant + pembrolizumab (NCT05082259) and a trial combining xevinapant with avelumab (NCT03270176). The fact that the radio- and chemo-sensitizing effect of IAP inhibitors are seen even in mice without an immune system suggests some of their effects might be independent of adaptive immunity, and we expect data from these last human trials will help disentangle the contribution of adaptive immunity on these drugs’ effectiveness.

Although the focus of our review is on the IAPs and their respective inhibitors, one should note that the IAPs are not the only possible target for pharmacological intervention in the apoptosis pathways.

As mentioned before and as outlined in Figure 2, the pro-survival protein B-cell lymphoma-2 (BCL-2), which regulates the permeability of the mitochondrial membrane and inhibits the triggering of the intrinsic pathway of apoptosis, has already been shown to be an effective target. BCL-2 (as well as other proteins of the same family) act upstream of caspase activation in the intrinsic pathway of apoptosis. The members of the BCL-2 family regulate mitochondrial permeability. Concretely, they inhibit the release of the pro-apoptotic molecules Smac and cytochrome C from the mitochondria into the cytoplasm. Because they share mechanisms of action with the endogenous BH3, those inhibitors are also known as BH3-mimetics.

Some authors classify such drugs as senolytics [18,43]. Venetoclax is a specific BH3-mimetic/BCL-2 inhibitor that has shown effectiveness in the treatment of several hematological malignancies and has been approved for clinical use [44]. Despite promising pre-clinical results in human solid tumors, no BH3 inhibitor has so far been effective for solid tumors, although they have been relatively understudied. Resistance to BH3 inhibitors against one specific protein in the BCL-2 family may develop as a result of the compensatory overexpression of the other proteins of the same family. This has been shown in some solid tumors, namely pediatric solid tumors, small-cell lung cancer, breast cancer, and melanoma [43]. Still, despite the disappointing results so far, further studies with this class of drugs may yet reveal a role in solid tumors. However, one should note that the IAP inhibitors reviewed in this paper act downstream of the BCL-2 family and, as such, may be superior in terms of effectiveness, at least in solid tumors. The concomitant use of a BH3-mimetic (venetoclax) and IAP inhibitor (tolinapant) has shown improved effectiveness in a pre-clinical model of T cell lymphoma [45]. It is not yet clear whether this drug combination will be safe and effective in clinical practice.

## 5. Smac-Mimetics and Other Strategies for Apoptosis Inhibition

A number of clinical trials with IAP inhibitors have been performed or are ongoing. Currently, the only IAP inhibitor that has shown positive results in a phase II trial is Xevinapant. The trial (NCT02022098) included 96 patients with locally advanced unresectable p16-negative SCHNC, randomized in a 1:1 ratio to either radiotherapy concomitant with high-dose cisplatin (100 mg/m^2^, 3 cycles) vs. radiotherapy concomitant with high-dose cisplatin and xevinapant. The fact that all patients were p16-negative, as well as the locally advanced stage at diagnosis, enriched the study sample for patients at high risk of tumor persistence or relapse after chemo- and radiotherapy. At three years, the risk of death or disease progression was reduced by 67% for xevinapant plus chemo-radiation (adjusted HR 0.33; 95% CI, 0.17–0.67; *p* = 0.0019). The risk of death was decreased by approximately half in the xevinapant arm compared with the placebo (adjusted HR 0.47; 95% CI, 0.27–0.84; *p* = 0.0101). Overall survival was prolonged within the xevinapant arm when compared to the control arm: median OS not reached (95% CI, 40.3-not evaluable) vs. 36.1 months (95% CI, 21.8–46.7) [20]. These are very promising results. Although toxicity was high (as expected in this treatment protocol), adding xevinapant did not increase any of the toxicities of either cisplatin or radiotherapy. Notably, neutropenia and mucositis were not increased [46] (which might suggest that prevention of apoptosis via the AIPs is not a clinically important mechanism in bone marrow stem cell and mucosal cell survival). No increase in late-onset toxicities was observed at 3 years, which is important in curative treatment, in which some patients are expected to have a high life expectancy after treatment. The success of this phase II trial led to the development of three phase III trials for head and neck cancer.

The subsequent phase III trial of TryllinX (NCT04459715) excluded patients with p16-positive cancers [21]. This choice is, in one sense, understandable, as these patients have, on average, a better prognosis in locally advanced disease, both in terms of disease-free survival and overall survival [47]. However, although p16-positive patients are the ones less likely to derive a large benefit from the addition of xevinapant to the standard chemo-radiation protocol, no increased early-onset or late-onset toxicities were detected in the phase II trial. Thus, this form of treatment escalation seems to be as safe as the standard chemo-radiation regimen. We argue that p16-positive patients should have been included in the TryllinX trial. As it stands now, even if the trial is positive, the question will linger on whether the addition of xevinapant is effective in this lower-risk population.

A new double-blind, randomized phase III trial (NCT05930938) is expected to start soon with the goal of comparing definitive chemo- and radiotherapy cetuximab + xevinapant with cetuximab plus radiotherapy in patients unfit for high-dose cisplatin. Unlike the previous trial, p16-positive patients can be included, as long as they have a high-risk stage, namely T3 N1-3 or T4 any N.

The XRAY VISION trial (NCT05386550) is a phase III study for the adjuvant treatment of resected SCHNC in high-risk patients ineligible for cisplatin, comparing radiotherapy alone with radiotherapy plus xevinapant. The trial is actively recruiting for a goal of 700 participants. Patient recruitment is expected until October 2027, and study completion is expected in December 2030.

The European Organisation for Research and Treatment of Cancer (EORTC) RAVINA trial (NCT05724602) is a multicenter, randomized, placebo-controlled, triple-blind, phase II study to determine the efficacy and safety of xevinapant with radiotherapy in patients 70 years old or older, which are ineligible for chemo-radiation with cisplatin with locally advanced head and neck squamous cell carcinoma of the oral cavity, oropharynx, hypopharynx, or larynx. RAVINA trial aims to test xevinapant as a safe and effective drug to escalate treatment in unfit patients, which are often excluded from other clinical trials. Unlike the TryllinX study, the RAVINA study will allow p16-positive patients (although it excludes low-risk p16-positive patients, namely those with T1-2N1 disease), which might pave the way for treatment escalation with xevinapant in this population. The estimated start date for the study is September 2023, with patient recruitment until July 2029.

These trials are notable for testing a new agent in the setting of locally advanced disease in which the patient is a candidate for curative treatment. In contrast, most clinical trials are performed first in the recurrent/metastatic disease and are only tested in the setting of localized disease after proving effectiveness in the recurrent/metastatic setting. However, with the unique mechanism of action of the inhibitors of the IAPs and their capability of reverting senescence and overcoming chemotherapy resistance, it is expected they will be particularly effective in increasing the disease-free interval and cure rates of at least some selected chemo- and/or radiotherapy protocols.

Other IAP inhibitors, such as birinapant and tolinapant, are currently being studied in phase I and phase II trials. Although out of the scope of this review, we call attention to the already mentioned trials combining IAP inhibitors with immunotherapy, namely the ASTEROID trial with tolinapant + pembrolizumab (NCT05082259) and a trial combining xevinapant with avelumab (NCT03270176). Although our focus has been more on solid tumors, tolinapant has ongoing trials in hematological malignant diseases (NCT05403450). In Table 1, we summarize the key clinical and pre-clinical studies related to the mechanisms of action and importance of the IAPs in cancer treatment, which we mentioned throughout this article.

## 6. Conclusions

Due to the way the IAPs inhibit apoptosis and given the apparent importance and ubiquity of IAP overexpression in tumors (in particular, XIAP), the inhibitors of apoptosis proteins might probably be useful as an adjuvant to cytotoxic chemotherapy and radiotherapy in a number of contexts. These drugs seem particularly suited in the curative setting when maximal cytoreduction is desired, and the minimization of survival of resistant cells is paramount.

Switching the cellular program from senescence (in which case cells could lie dormant for arbitrarily large amounts of time) towards apoptosis might be one of the keys to achieving a complete response in both localized disease and distant micrometastases. Since the phase II trial of xevinapant was successful, while previous birinapant trials in the setting of metastatic disease were not, one might think exploiting the Smac mimetics in potentially curative treatments.

The effectiveness of xevinapant plus cisplatin plus radiotherapy naturally suggests one might attempt in vitro and in vivo trials of xevinapant plus chemoradiation with cisplatin for cervical cancer, as it might increase the pro-apoptotic effects of DNA damage induced via both cisplatin and radiation. Pre-clinical data from rectal cell lines, human tumors, and murine models have implicated both XIAP [47] and cIAP1/2 [36] in resistance to radiation and chemo-radiation in rectal cancer. Based on this preclinical evidence, a phase Ib clinical trial with the IAP inhibitor tolinapant plus preoperative chemo-radiation is estimated to start in late 2023 (NCT05912075). We hypothesize that the use of Smac mimetics could be key to increasing the rate of pathological complete responses of neoadjuvant radiotherapy or fluoropyrimidine-based chemo-radiation for rectal cancer, allowing for a watch-and-wait protocol instead of the often mutilating pelvic surgery required for tumor excision.

Despite 50 years of targeted therapy, many patients are still underserved by these newer kinds of drugs in the setting of locally advanced or metastatic disease. However, a majority of these patients will be candidates for classical cytotoxic and cytostatic chemotherapy and/or radiotherapy. As a new class of drugs, the IAPs seem well positioned to increase the effectiveness of both these older drugs and radiotherapy, increasing the probability of a cure when a cure is possible, and maybe overcoming drug resistance when a cure is not possible. We eagerly await developments from the ongoing phase III and phase II trials as well as new preclinical data that might show further uses for this remarkable class of drugs.

## Figures and Tables

**Table 1:** This table summarizes the main clinical and pre-clinical studies discussed throughout this review related to the effectiveness and mechanism of action of the IAP inhibitors. The table does not aim to represent all pre-clinical research available on this class of drugs, which at the time of writing is quite extensive. FOLFOX—chemotherapy regimen of 5-fluorouracil plus oxaliplatin; mFOLFIRINOX—modified regimen of 5-fluorouracil, irinotecan plus oxaliplatin; CAPOX—capecitabine plus oxaliplatin.

Drug	Study Reference	Phase	Study Population	Status	Summary
None	[48]	Preclinical	Human rectal carcinoma cell lines	-	XIAP seems to mediate radio-resistance in rectal cancer cell lines
None	[38]	Preclinical	Cancer gene expression data from open data repositories	-	Bioinformatic analysis shows that high XIAP expressions correlate with markers of chemoresistance in breast cancer.
Tolinapant (+FOLFOX)	[36]	Preclinical	Human and murine rectal carcinoma organoids	-	cIAP1/2 seems to mediate chemo-resistance in rectal cancer, and tolinapant can help overcome chemoresistance
Tolinapant (+venetoclax)	[45]	Preclinical	In vitro cells of T cell acute lymphoblastic leukemia	-	Tolinapant sensitizes apoptosis of T cell leukemia cells induced via venetoclax or dexamethasone.
Tolinapant (+pembrolizumab)	ASTEROID trial (NCT05082259)	Phase I	Multiple metastatic tumors	Ongoing, recruitment active	No results available.
Tolinapant (+Decitabine/Cedazuridine)	NCT05403450	Phase I/II	Patients with relapsed/Refractory Peripheral T-cell Lymphoma	Ongoing, recruiting active	No results available
Tolinapant (+capecitabine), followed or preceded by mFOLFIRINOX or CAPOX	NCT05912075	Phase Ib	Patients with locally advanced rectal cancer	Not yet recruiting	No results available
Xevinapant (+avelumab)	NCT03270176	Phase I	Multiple metastatic tumors including non-small cell lung cancer	Ongoing, recruitment in progress	No results available.
Xevinapant (+high-dose cisplatin + radiation)	NCT02022098 [20]	Phase II	Locally advanced p16-negative head and neck cancers	Completed	At three years, the risk of death or disease progression was reduced by 67% for xevinapant plus chemo-radiation (adjusted HR 0.33; 95% CI, 0.17–0.67; *p* = 0.0019). The risk of death was decreased by approximately half in the xevinapant arm compared with placebo (adjusted HR 0.47; 95% CI, 0.27–0.84; *p* = 0.0101). Overall survival was prolonged.
Xevinapant (+high-dose cisplatin + radiation)	TryllinX (NCT04459715) [21]	Phase III	Locally advanced p16-negative head and neck cancers	Ongoing, recruitment completed	No results available.
Xevinapant (+high-dose cisplatin + radiation)	XRAY VISION (NCT05386550)	Phase III	Operable head and neck cancers in the neoadjuvant and adjuvant setting	Ongoing, actively recruiting	No results available.
Xevinapant (+radiation)	RAVINA (NCT05724602)	Phase III	Locally advanced high-risk p16-negative and p16-positive patients unable to tolerate high-dose cisplatin	Not yet recruiting	No results available.

## Data Availability

This manuscript does not contain any original data.
